# Improving CSI Prediction Accuracy with Deep Echo State Networks in 5G Networks

**DOI:** 10.3390/s20226475

**Published:** 2020-11-12

**Authors:** Tommaso Pecorella, Romano Fantacci, Benedetta Picano

**Affiliations:** Department of Information Engineering, University of Florence, 50139 Firenze, Italy; tommaso.pecorella@unifi.it (T.P.); romano.fantacci@unifi.it (R.F.)

**Keywords:** channel state information, recurrent neural networks, time series forecasting

## Abstract

The forthcoming fifth-generation networks require improvements in cognitive radio intelligence, going towards more smart and aware radio systems. In the emerging radio intelligence approach, the empowerment of cognitive capabilities is performed through the adoption of machine learning techniques. This paper investigates the combined application of the convolutional and recurrent neural networks for the channel state information forecasting, providing a multivariate scalar time series prediction by taking into account the multiple factors dependence of the channel state conditions. Finally, the system performance has been analyzed in terms of prediction accuracy expressed as absolute deviation error and mean percentage error, in comparison with an alternative machine learning method recently proposed in the literature with the aim at solving the same prediction problem.

## 1. Introduction

The emergence of the need for new network technologies and architectures has been launched by the diffusion of novel services and applications demanding high data rates and reliability, low latency, and congestion levels. Augmented reality, automatic driving, and tactile internet are only a few of the examples of fifth-generation (5G) applications. Furthermore, new era applications require remarkable improvements with regards to the intelligence core of the network, giving rise to self-organizing ecosystems able to contextualise awareness in all its different forms and facets. In fact, one of the most challenging issues in 5G networks is the empowering of the learning processes, recreating on devices actual human neural network behavior to obtain effective solutions to new era networks problems.

From previous network generations, the analysis and design of innovative technologies and methodologies to move towards wireless systems able to optimally exploit radio resources and actuate proper decision policies has gained attention. During the years, cognitive radio (CR) approaches have played a crucial role, aiming at designing context-driven strategies. In this regard, the advent of the 5G networks, which involve the usage of the millimeter wave (mmWave) band has resulted in a large variety of obstacles such as pathloss, blockage, and high oxygen absorption [1]. All these factors increase the complexity of detecting actual channel conditions and signals characteristics. Under these circumstances, the channel state information (CSI) represents one of the most important concepts in the wireless networks [2,3,4], providing a performance metric for the quality of the radio channel. The CSI describes the physical layer parameters arranged for wireless communication systems [2], being crucial in many decision making areas such as radio resource allocation [5], interference management [6], and so on. Consequently, an accurate value of the CSI plays a role of paramount importance in a wide range of practical applications, while inaccuracy on the CSI prediction value leads inevitably to substantial degradation on communication effectiveness.

Generally speaking, the channel prediction is an impressive approach to obtain accurate CSI values. Differently, during years, many CSI estimation methods have been proposed [2], from the maximum-likelihood estimation [2,7], up to the minimum mean squared error estimation [2,8], passing through the least square estimation [2,9].

Typically, these methods involve matrix operations as the matrix inversion or the eigenvalue decomposition, resulting in procedures characterized by high computational costs. Due to such hardness in channel condition knowledge or the presence of remarkable overhead costs associated with the traditional channel state estimation methods, the design of novel strategies has become a necessity in reference to upcoming 5G networks. In fact, 5G environments have to face highly complex scenarios, in which CSI prediction is a large-scale channel problem, due to the presence of massive multiple-input-multiple-output (MIMO), orthogonal frequency division multiplexing (OFDM), and mmWave communications [2]. As consequence, the matrix operations involved in the traditional CSI estimation methods, applied to 5G contexts, bring large-scale matrix operations with significant computational complexity and overheads, therefore becoming unviable approaches implying long convergence time [2].

Recently, the machine learning (ML) techniques have gained attention for their application in numerous research fields such as channel prediction [10], detection [11], modulation [12], and so on. Therefore, the ML provides modules and frameworks suitable to be contextualized to the CR environments, especially those in which several factors are involved. Indeed, the CSI parameter depends on a multitude of factors interrelated to each other as humidity, temperature, time, location, etc. [2], which contribute to the definition of the CSI. In this sense, scalar time series analysis and prediction based on the ML may represent a concrete strategy to provide accurate CSI forecast.

This paper proposes the application of the ML principles to the CSI prediction, by formulating that problem in terms of scalar time series forecasting [13].

Overall, the class of the prediction problems can be classified into short-, medium-, and long-term forecasting. In the first one, the predictions are performed over horizons from a few minutes up to a few days ahead, and the second one deals with time horizons from few days to few months ahead. Finally, predictions from quarters to years ahead [14] are named long-term forecasting. It is important to highlight that typically, medium and long-term forecasting involve the prediction about risk management and profitability planning. Differently, short-term forecasting is applied to traffic demands and mobility prediction [14,15]. Short-term forecasting has been extensively studied in the literature, and many different methods have been proposed [13,15]. Therefore, the aims of the paper are summarized as

- Design and development of an ML-based framework involving the usage of the convolutional neural networks (CNNs) and the recurrent neural network (RNN) to predict the CSI behavior;
- Extensive numerical simulations to provide the performance evaluation analysis as regards the prediction accuracy expressed as mean absolute deviation error and mean percentage error between the actual CSI value and those predicted;
- Testing of the proposed framework under different application scenarios, i.e., outdoor and indoor conditions, in comparison with the deep learning strategy proposed in [2] and that presented in [16].

The rest of the paper is organized as follows. Section 2 presents an in-depth review of the related literature, while in Section 3 the system model and the problem formulation is detailed. Section 4 presents the technique adopted to reach the main objective of the paper. Validations of the proposed scheme are presented in Section 5, and conclusions are drawn in Section 6.

## 2. Related Works

The CSI prediction problem has been largely studied and, during the years, different approaches have been pursued. Recently, the involvement of ML techniques for estimating the CSI has gained momentum. Authors in [17] address the problem of the short-term forecasting of the fading channel. In fact, they propose the application of both the machine learning and the Savitzky–Golay filter to increase the prediction accuracy. The paper [18] focuses on the application of the ML to the adaptive coding and modulation, considering satellite networks environments. More specifically, in [18] the prediction is performed on the signal-to-noise ratio series, and the remarkable forecasting accuracy derived by the application of the ML approach in comparison with the traditional alternatives. Furthermore, in [19], the authors expose the channel fading prediction for addressing the CSI aging problem to increase the network throughput and spectral efficiency. Differently, the sparse Bayesian linear regression combined with the support vector machine approach has been proposed in paper [20], whose main objective is the joint CSI prediction and the dynamic radio frequency slicing, considering the software-defined networks and the MIMO systems. Massive MIMO contexts have been also considered in [21], in which the ML has been applied to lower the overhead due to the downlink channel estimation. The proposed scheme consists of the combined usage of the linear regression and the support vector regression in ML. More in detail, the authors in [21] have involved realistic CSI samples to train the regression model. Then, the CSI antennas are online estimated by inputting the output data derived by the regression model. Similarly, the overheads due to the orthogonal pilot based estimation has been widely studied in paper [16], by designing an ML oriented scheme focusing on the channel time correlations analysis. In fact, the framework proposes the application of a CNN together with an auto-regressive model or an RNN auto-regressive network with exogenous inputs.

Differently, in the paper [22] highly mobile vehicular environments are considered. The authors affirm that, due to the almost identical propagation conditions, a deep learning approach may acquire and analyze the non-linear temporal correlation characterizing subsequent CSI samples, without the side effect of a remarkable overhead. In addition, in [22] a resource allocation for the network slicing problem is presented, by assuming vehicular users demanding enhanced mobile broadband (eMBB) and ultra-reliable low latency (URLLC) traffic slices. Finally, the CSI estimation within the free space optical communication networks is proposed in the paper [23], where also in this case an ML approach is pursued. Specifically, the authors involve the usage of both the maximum likelihood estimation and the Bayesian estimation methods, to estimate the channel coefficients of interest for the considered scenario.

## 3. Problem Statement

The CSI forecasting problem has been largely studied and it strongly correlated to the power attenuation characterizing the radio signals when transmitting over a distance. In fact, generally speaking, a radio channel is affected by phenomenons as scattering, fading, or path loss [2]. The CSI represents a parameter providing a measurement of the combined effect of all these factors. As is well known from literature, the path loss can be expressed as follows

(1) $$\mathrm{PL}=10\log_{10}\frac{P_{\mathrm{tx}}}{P_{\mathrm{rx}}},$$

where $P_{\mathrm{tx}}$ and $P_{\mathrm{rx}}$ are the transmitting and the receiving power, respectively. Equivalently, Equation (1) can be expressed as a function of the distance as [2]

(2) $$-10\log_{10}G{\left(\frac{\lambda }{4\pi d}\right)}^{2},$$

in which the term *G* represents the product of the antenna field radiation pattern [2], *d* is the distance between a transmitter and the corresponding receiver, while $\lambda =3\times 10^{8}/f$ represents the wavelength and *f* the frequency. Wireless communications, in addition to being affected by path loss, suffer also from scattering and fading. Although the scattering is due to electromagnetic wave propagation, the fading is mainly dependent on the frequency and it is related to the obstacles and multi-paths propagation [2]. Therefore, all these factors make it challenging to provide an accurate CSI prediction. This paper takes into account a MIMO system, in which multiple antennas at the transmitter and receiver are used. MIMO exploits the multiple antennas to perform the spatial dimension in addition to the time and frequency ones, avoiding changes in the bandwidth requirements of the system. More specifically, this paper focuses on a slowly-fading MIMO channel model, in which the channel state remains quasi-static within a fading block [24].

Furthermore, it is important to stress that we have that the CSI value results from a wide range of factors, such as weather, location, frequency band, time, and so on [2]. Considering a Wi-Fi channel and OFDM modulation, the raw CSI measure consists of complex numbers whose amplitude and phase are influenced by the propagation conditions of the communication that usually give rise to an amplitude attenuation and phase shift for the received signal. Such values depend on the frequency band used. For more details about the CSI extraction in Wi-fi MIMO systems in practice, we refer to [25,26]. It is important to highlight that this paper, inspired by [2], considers the CSI, as resulting from the combination of the following factors

- *Frequency band*. We consider a set of frequency bands for 5G denoted by $\left\{f_{1},\cdots ,f_{N}\right\}$, hereafter used interchangeably with channels.
- *Location*. Due to the fluctuation of the factors previously introduced, different regions within the base station coverage may experience different CSI. Indeed, the locations are represented by $\left\{l_{1},\cdots ,l_{M}\right\}$, where $l_{m}$ is the *m*-th location, expressed as sub-region.
- *Time*. Due to the fact that the atmosphere density changes during different hours in a day and seasons [2], we slotted the time as $\left\{\tau_{1},\cdots ,\tau_{P}\right\}$, where $\tau_{i}$ represents the time slot *i*.
- *Weather*. The weather massively influences the CSI. In fact, as modeled in [2], we considered the following weather levels
  1. sunny;
  2. lightly cloudy;
  3. cloudy;
  4. lightly rainy;
  5. medium rainy;
  6. heavy rainy;
  7. light snow;
  8. medium snow;
  9. heavy snow.

Then, the corresponding weather set is denoted by $W=\{w_{1},\cdots ,w_{R}\}$, in which $w_{r}$ represents the corresponding weather level. As consequence, in this paper we consider the *CSI sample*, hereafter referred to STATE, given as input to the proposed learning strategy, having the following format

(3) $$STATE=(f_{n},l_{m},\tau_{p},w_{r},CSI),$$

in which $f_{n}$ represents the band, $l_{m}$ the location, $\tau_{p}$ the time, and $w_{r}$ the weather level, as previously detailed. The main objective of the paper is short-term CSI forecasting.

## 4. Forecasting Strategy

Recently, CNNs have been largely applied in many image processing and recognition problems. In this paper, we exploit a framework architecture yet presented in literature based on the CNNs [2], and then we combined such neural network architecture to a particular type of recurrent neural network (RNN) particularly suitable for its low complexity, named echo state network (ESN). The RNNs allows the exploitation of the correlation between consecutive samples within the time series.

**Figure 1:**
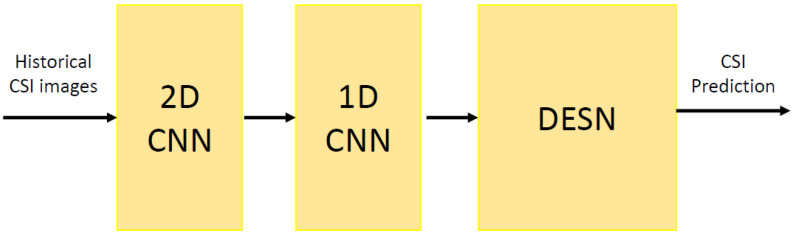
The forecasting framework.

As detailed in [2], the CNN based architecture consists of a 2D CNN and a cascade 1D CNN. In fact, the first 2D convolution module, as depicted in Figure 1 the CNN modules take as input the historical CSI images, i.e., the images of the data collected along time on which the training is performed. More specifically, the 2D CNN is responsible for the frequency extraction, in which there is a composed of a set of filters in parallel and then a set of weights. Consequently, the state representative vector is extracted by the 1D network from the frequency representative vectors [2]. Therefore, the last component of the proposed framework is the ESN module. The ESN is a particular RNN, based on the Reservoir Computing (RC) paradigm [27,28] which, recently has been considered an effective approach for efficiently training the RNNs. Despite many instances of the RC methodology have been presented in the literature [29], the ESN [30] is one of the most known models [31,32,33,34,35]. Roughly speaking, the ESNs belong to a subclass of the RNN, named as the recurrent randomized neural networks [36], whose state dynamics are implemented by an untrained recurrent hidden layer [37].

The Deep Echo State Network (DESN) [38] emerged as a promising model for efficiently training deep neural networks for temporal data [37]. The DESN is mainly composed of the following characteristics [37]

- a dynamical reservoir component, transforming the input history into a state representation;
- a feed-forward readout component, which computes the output.

**Figure 2:**
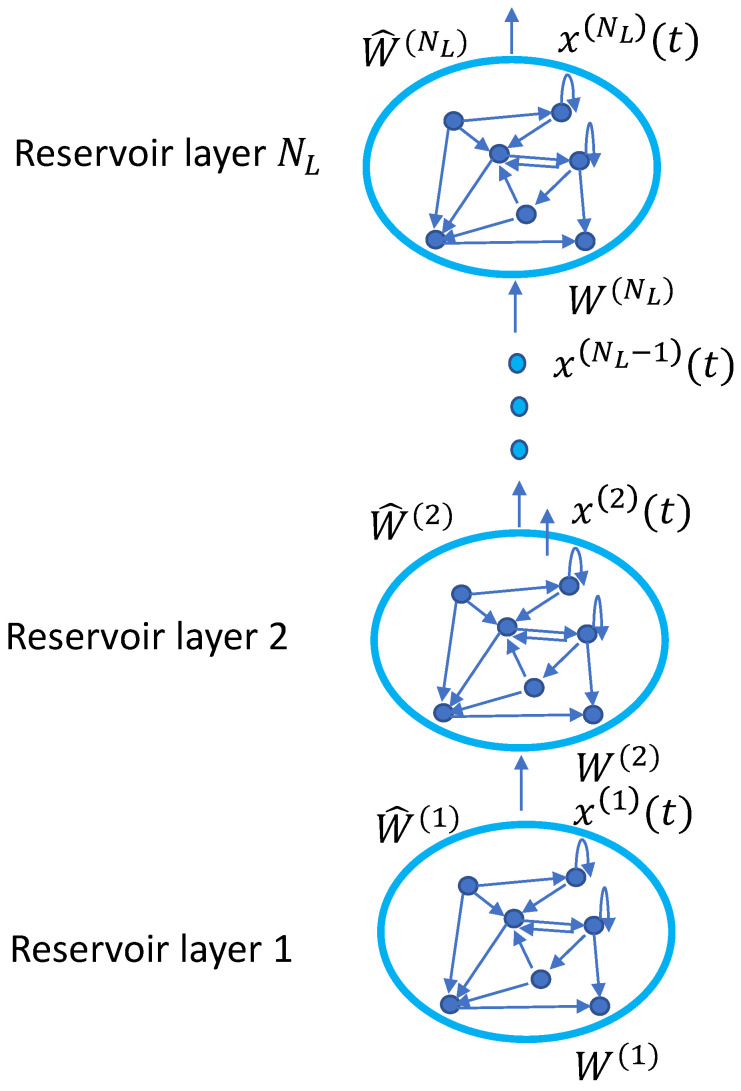
Reservoir architecture.

As widely discussed in the literature [37], the reservoir of a DESN is characterized by a hierarchy of cascade recurrent layers, where the output of each layer represents the input for the next one, as illustrated in Figure 2. At each time step *t*, let $N_{U}$ be the external input dimension, and let $N_{L}$ be the number of reservoir layers. Furthermore, each reservoir layer has $N_{R}$ recurrent units. Then, the external input at time *t* is defined by $u\left(t\right)\in R^{N_{U}}$, while $x^{\left(i\right)}\left(t\right)\in R^{N_{R}}$ represents the state of the reservoir layer *i*-th at time slot *t*. At each time slot *t*, the global state of the DESN, i.e., the state in all the reservoir layers is expressed by

(4) $$x\left(t\right)=\left(x^{\left(1\right)}\left(t\right),\cdots ,x^{\left(N_{L}\right)}\left(t\right)\right)\in R^{N_{R}N_{L}}.$$

In formal terms, the reservoir dynamics for the first layer is described as in (5), while

(5) $$x^{\left(1\right)}\left(t\right)=F\left(u\left(t\right),x^{\left(1\right)}\left(t-1\right)\right)=\left(1-a^{\left(1\right)}\right)x^{\left(1\right)}\left(t-1\right)+f\left(W^{\left(1\right)}u\left(t\right)+{\hat{W}}^{\left(1\right)}x^{\left(1\right)}\left(t-1\right)\right),$$

the *i*-th update is given by (6), in which $W^{\left(1\right)}$ and $W^{\left(i\right)}$ are the input and the inner weight matrix, respectively. More in depth, $W^{\left(i\right)}$ represents the weight matrix for the connection of the (i−1)-th inner layer to the *i*-th. Similarly, ${\hat{W}}^{\left(i\right)}$ defines the recurrent weights matrix for layer *i*, while $a^{\left(i\right)}$ is the leaking rate and f is the activation function here selected as the hyperbolic tangent.

(6) $$x^{\left(i\right)}\left(t\right)=F\left(x^{\left(i-1\right)}\left(t\right),x^{\left(i\right)}\left(t-1\right)\right)=\left(1-a^{\left(i\right)}\right)x^{\left(i\right)}\left(t-1\right)+f\left(W^{\left(i\right)}x^{\left(i-1\right)}\left(t\right)+{\hat{W}}^{\left(i\right)}x^{\left(i\right)}\left(t-1\right)\right),$$

Accordingly to the RC framework, the reservoir parameters, i.e., the matrices weights, are left untrained and constrained through stability conditions [37].

In reference to the computation of the output, typically the global state of the network feeds, at each time slot *t*, the output layer. Consequently, the readout gives different weights to the dynamics developed at different layers, favoring the diversity exploitation due to the representations in the cascade reservoir.

Therefore, by supposing the output size given by $N_{Y}$, at time slot *t* the output can be expressed as [37]

(7) $$y\left(t\right)=W_{\mathrm{out}}x\left(t\right)=W_{\mathrm{out}}\left(x^{\left(1\right)},\cdots ,x^{\left(N_{L}\right)}\right),$$

in which $W_{\mathrm{out}}\in R^{N_{L}\times N_{R}N_{L}}$ is the readout weight matrix. To predict the CSI value, we have to transform the input STATE into a format processable by the proposed CNN based strategy. Therefore, the approach proposed in [2], in which the CSI is represented as a pixel image I, has been pursued resulting in

(8) $$I=[C_{1},\cdots ,C_{l}],$$

in which $C_{i}$ represents the *information segment* of the CSI, in accordance with [2]. At this point, the input CSI image is ready to be processed by the CNN, in which the convolutional filters are applied to the image giving rise to the feature image $I^{*}$, which represents the collection of the outputs stem by the application of the filters. Once the convolutional filters are combined together, the representative vectors are build by concatenating the information segment. Then, the representative vectors, each of which corresponds to a single frequency, are concatenated to obtain the state representative matrix of the considered time slot. Finally, the 1D CNN is applied to catch the temporal features from the data matrix. Finally, the DESN is applied to obtain the CSI prediction considering the minimization of the mean percent error (MAPE), defined as

(9) $$\mathrm{MAPE}=\frac{1}{M}\sum_{i=1}^{M}\left|\frac{{\hat{I}}_{i+\delta }-I_{i+\delta }}{I_{i+\delta }}\right|\cdot 100,$$

where *M* represents the number of the samples in test data, and ${\hat{I}}_{i+\delta }$ and $I_{i+\delta }$ are the actual and the predicted values at time i+δ, where δ expresses the increment on the time horizon of the predictive strategy. In addition, to provide an exhaustive analysis, we have also considered the mean absolute deviation (MAD) given by

(10) $$\mathrm{MAD}=\frac{1}{M}\sum_{i=1}^{M}\left|{\hat{I}}_{i+\delta }-I_{i+\delta }\right|.$$

It is important to note that metric (10) highlights the variability of the forecasting error, while (9) expresses the error in terms of percentage on the actual data.

The proposed procedure acts throughout two phases: the online and the offline stages. The offline module corresponds to the learning phase, performed over the massive volume data collected over time, to catch the CSI behavior trend over time. In this framework stage, the stochastic descent gradient method is applied, together with the backpropagation technique, aiming at training the matrix weights *W*. Then, the prediction is improved during the online phase, in which the new CSI measurements are used as feedback for the proposed neural network framework. The online phase update is periodic and the model is updated following the standard law given by [2]

(11) W=W−α∇W,

where α expresses the learning rate. For the offline phase, α has been selected within the interval [−0.1,0.1], while the online phase exhibits α=0.1. Finally, to perform the prediction, we have assumed a 2×2 filter for the 2D CNN, and a 3×1 filter for the 1D CNN. Then, the DESN has been set considering 128 hidden nodes, in which the connection has been randomly established.

## 5. Numerical Results

In this paper, we analyzed two sets of data related to the nonlinear CSI time series corresponding to the realistic conditions of indoor and outdoor environments by collecting actual CSI samples on the campus with regards to the outdoor data, and within a building for the indoor scenario. Furthermore, it is important to highlight that the outdoor environment considers free space conditions, while the indoor gathering procedure has been performed in presence of several obstacles such as desks, chairs, walls, doors, and other ordinary office objects. The data have been collected by using two Nexus and three Raspberry Pi devices. Then, the data sets collected contain over 1500 non-duplicating samples, in which two-tiers of data are used for offline learning and the remaining data are used for validating the procedure. In fact, the obtained performance results derived by the usage of the collected CSI samples for both the framework training and testing, properly split among the training and testing sets. Furthermore, to provide an exhaustive analysis, we compare the proposed DESN method with the OCEAN scheme recently proposed in [2] and the method proposed in [16], hereafter referred to as the LSTM.

### Discussion

**Figure 3:**
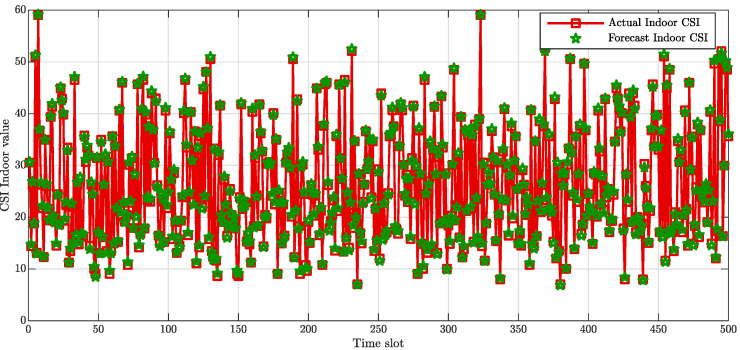
Indoor channel state information (CSI) amplitude value comparison in dB.

**Figure 4:**
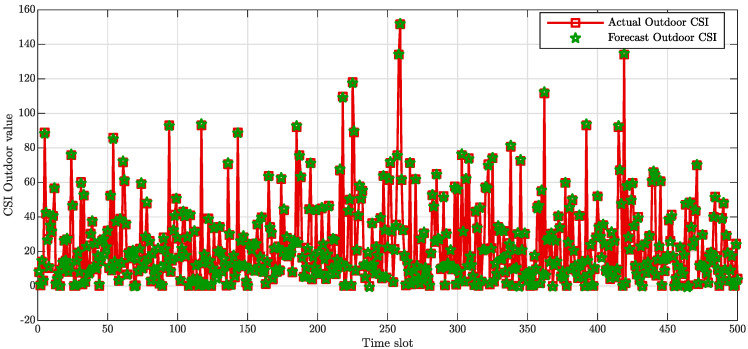
Outdoor CSI amplitude value comparison in dB.

The comparison between the collected data and the data predicted are reported in Figure 3 and Figure 4, with regards to the indoor and outdoor conditions scenario, respectively. Figure 3 exhibits the closeness between the actual data collected within a building and the predicted values. Similarly, Figure 4 shows that the forecast CSI are remarkably close to those collected within an outdoor environment.

**Figure 5:**
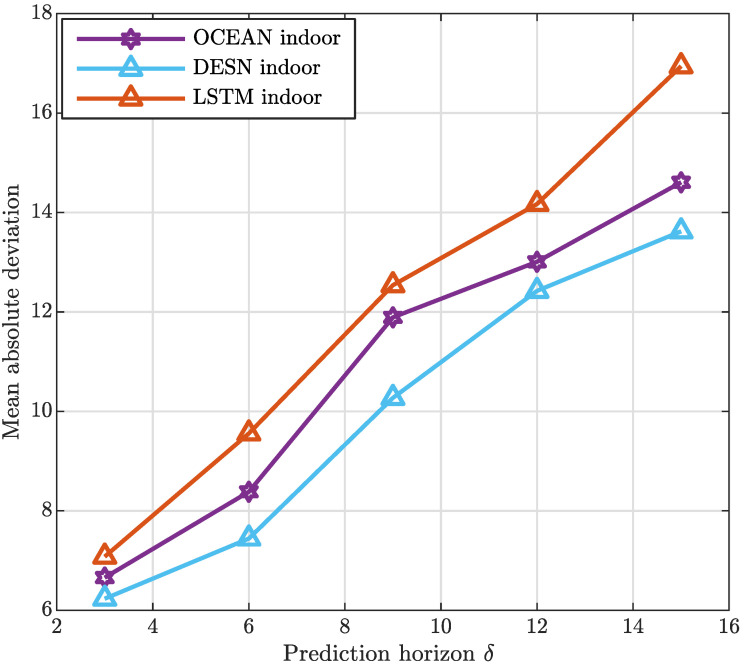
Indoor mean absolute deviation.

**Figure 6:**
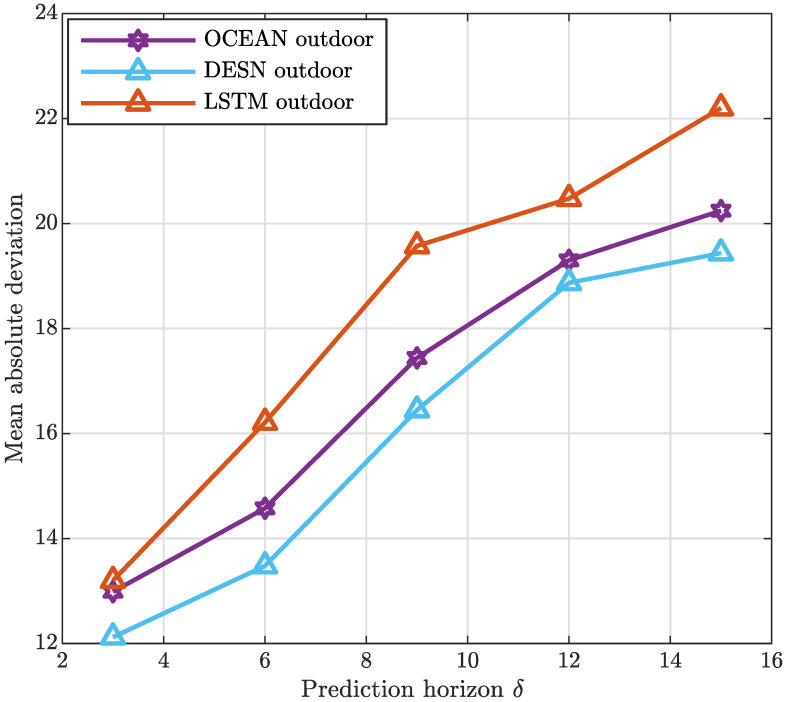
Outdoor mean absolute deviation.

**Figure 7:**
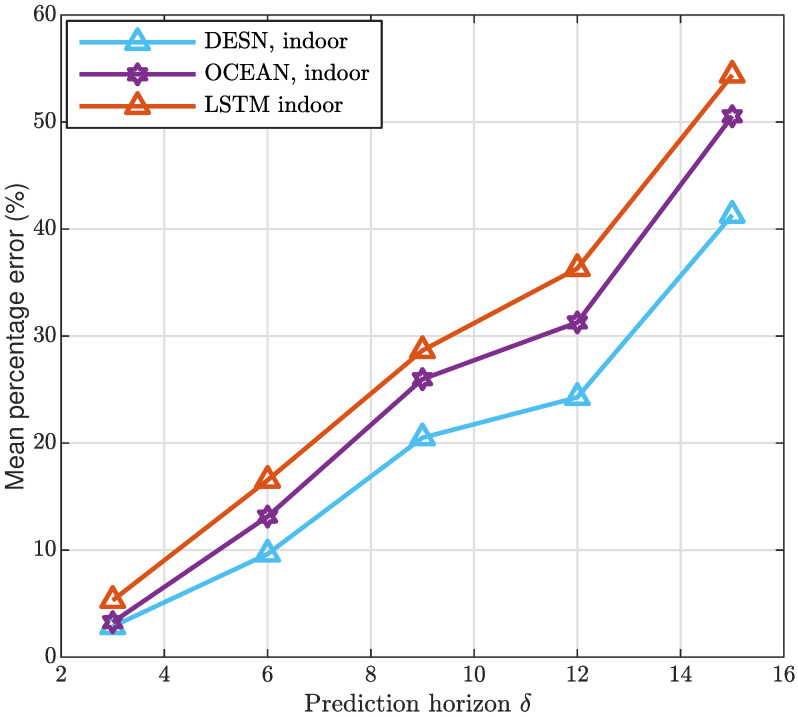
Indoor mean absolute percentage error.

**Figure 8:**
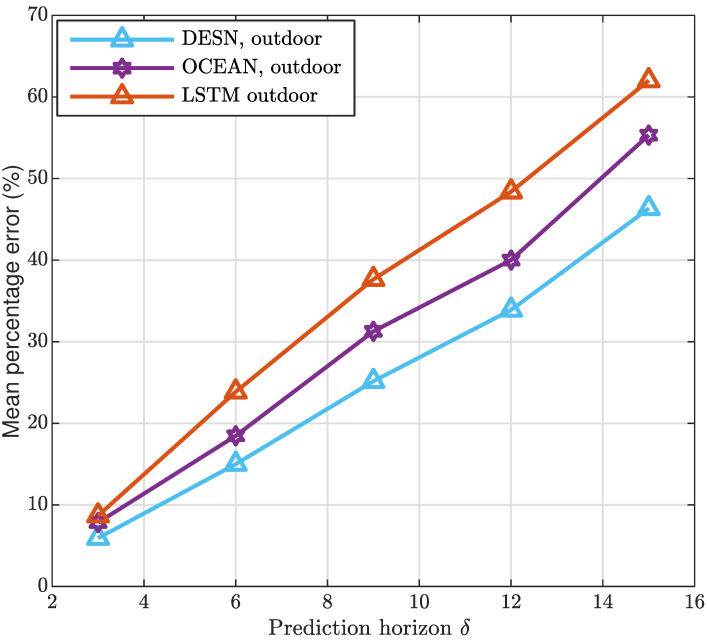
Outdoor mean absolute percentage error.

Aiming at analyzing the performance of the strategies taken into account, the MAD has been represented in Figure 5 and Figure 6 considering, also in this case, both the indoor and outdoor application contexts.As it is clearly evident from both the Figures, the prediction accuracy is greater when the proposed algorithms are applied. In addition, it is important to highlight that one slot is assumed equal to 4 ms. Furthermore, in all the strategies the MAD grows by increasing δ hereafter expressed in milliseconds, and denoting the time horizon considered to perform the prediction. It is a direct consequence of the complexity in predicting system dynamics over long interval times. To extensively analyze the framework designed, Figure 7 and Figure 8 depict the MAPE metric trend, for the indoor and outdoor cases, respectively. The results confirm the good performance of the proposed approach in comparison with the alternatives taken into account.

In conclusion, the proposed DESN approach provides a valuable framework for short term prediction, within both the indoor and outdoor system conditions.

## 6. Conclusions

This paper has dealt with the CSI prediction problem, by proposing a forecasting strategy combining both the CNNs and the RNNs approaches and providing a method for predicting the CSI at the next time slot. This paper improves the CNN-based strategy recently proposed in literature by [2] and overcomes that proposed in [16].

Furthermore, the validation of the proposed scheme has been provided considering both the indoor and outdoor conditions, and the forecasting performance has been depicted in terms of mean forecasting errors to express the prediction accuracy. Finally, the CSI prediction accuracy resulting from the application of the proposed framework has been compared with that obtained with the strategy proposed in [2] and [16]. As it is evident to note from the simulation results, the proposed strategy overcomes the performance reached by applying the methods presented in [2,16], and constitutes a suitable prediction approach for the 5G networks. Such remarkable performance is due to the introduction of a further deep learning phase given by the DESN in comparison to the alternatives taken into account. In fact, the DESN, belonging to the RNN class, is particularly suitable in catching the temporal patterns characterizing data, resulting in a better fit of the data behavior.
